# Crystal Structure of a Putative Cytochrome P450 Alkane Hydroxylase (CYP153D17) from *Sphingomonas* sp. PAMC 26605 and Its Conformational Substrate Binding

**DOI:** 10.3390/ijms17122067

**Published:** 2016-12-09

**Authors:** Chang Woo Lee, Sang-Cheol Yu, Joo-Ho Lee, Sun-Ha Park, Hyun Park, Tae-Jin Oh, Jun Hyuck Lee

**Affiliations:** 1 Unit of Polar Genomics, Korea Polar Research Institute, Incheon 406-840, Korea; justay@kopri.re.kr (C.W.L.); psh@kopri.re.kr (S.-H.P.); hpark@kopri.re.kr (H.P.); 2 Department of Polar Sciences, University of Science and Technology, Incheon 406-840, Korea; 3 Department of BT-Convergent Pharmaceutical Engineering, Sunmoon University, Asansi 336-708, Korea; ysc9056@naver.com (S.-C.Y.); shadowjhl@empas.com (J.-H.L.)

**Keywords:** cytochrome P450, substrate binding assay, crystal structure, *Sphingomonas* sp., X-ray crystallography

## Abstract

Enzymatic alkane hydroxylation reactions are useful for producing pharmaceutical and agricultural chemical intermediates from hydrocarbons. Several cytochrome P450 enzymes catalyze the regio- and stereo-specific hydroxylation of alkanes. We evaluated the substrate binding of a putative CYP alkane hydroxylase (CYP153D17) from the bacterium *Sphingomonas* sp. PAMC 26605. Substrate affinities to C10–C12 n-alkanes and C10–C14 fatty acids with *K*_d_ values varied from 0.42 to 0.59 μM. A longer alkane (C12) bound more strongly than a shorter alkane (C10), while shorter fatty acids (C10, capric acid; C12, lauric acid) bound more strongly than a longer fatty acid (C14, myristic acid). These data displayed a broad substrate specificity of CYP153D17, hence it was named as a putative CYP alkane hydroxylase. Moreover, the crystal structure of CYP153D17 was determined at 3.1 Å resolution. This is the first study to provide structural information for the CYP153D family. Structural analysis showed that a co-purified alkane-like compound bound near the active-site heme group. The alkane-like substrate is in the hydrophobic pocket containing Thr74, Met90, Ala175, Ile240, Leu241, Val244, Leu292, Met295, and Phe393. Comparison with other CYP structures suggested that conformational changes in the β1–β2, α3–α4, and α6–α7 connecting loop are important for incorporating the long hydrophobic alkane-like substrate. These results improve the understanding of the catalytic mechanism of CYP153D17 and provide valuable information for future protein engineering studies.

## 1. Introduction

Cytochrome P450 enzymes (CYPs) are heme-containing proteins that catalyze the insertion of oxygen into a wide variety of substrates [1,2]. Although many different CYPs have been studied, alkane hydroxylases are among the most widely identified and characterized [3]. Hydroxylation of alkanes is important for their degradation or further metabolism, and chemical synthesis of hydroxylated alkane molecules is very difficult and costly [4]. Recently, CYP alkane hydroxylases have gained attention because of their commercial applications [5].

The CYP2E, CYP4B, CYP52, CYP86, and CYP153 families are known to hydroxylate n-alkanes or fatty acids in both eukaryotes and prokaryotes [6,7,8,9,10]. Among them, CYP2E, CYP4B, CYP52, and CYP86 are eukaryotic CYPs; these enzymes are membrane-bound proteins, limiting their structural characterization and making it difficult to use them in engineered pathways. Several bacterial CYPs in the CYP153 family have been characterized [11,12]. In 2012, the crystal structure of CYP153A7 (P450pyr) was determined [13]. In addition, researchers have attempted to engineer enzymes from the CYP101 and CYP102 families for alkane hydroxylation. For example, P450 BM-3 from *Bacillus megaterium*, a well-characterized CYP102 enzyme that functions as a fatty acid monooxygenase, has been used for this purpose [14]. To create useful oxidation biocatalysts for alkane compounds, P450 BM-3 enzyme was engineered to hydroxylate linear alkanes by directed evolution with mutagenesis [15]. In 2016, the structure of CYP153A from *Marinobacter aquaeolei* (CYP153A_M.aq_) was solved with and without its 12-hydroxydodecanoic acid ligand. The authors found that CYP153A_M.aq_ has terminal hydroxylase activity and that the A231G single mutation induced three-fold higher activity by increasing the flexibility of the substrate binding region [16]. Previous studies also demonstrated that *Sphingomonas* sp. HXN-200 bacteria contain hydrocarbon-degrading enzymes [17,18]. The results of these studies suggest that *Sphingomonas* sp. possesses CYP alkane hydroxylases with high alkane hydroxylation activity.

In the current study, we evaluated CYP153D17 (NCBI Reference Sequence, WP_010183267.1) from the endosymbiotic bacterium *Sphingomonas* sp. strain PAMC 26605. This bacterium was isolated from Arctic lichens found on the Svalbard Islands, Norway [19]. In addition, we determined the crystal structure of CYP153D17 at 3.1 Å resolution in complex with a co-purified alkane-like compound. Comparison of homologous CYP structures revealed a unique rearrangement of the active-site loop regions for adaptation of the long hydrophobic alkane-like compound near the heme iron in the active site. Here, we describe the structural determination of CYP153D17 and comparison of this structure with structural and sequential counterpart CYPs to improve understanding of the structural basis of conformational changes due to substrate binding.

## 2. Results and Discussion

### 2.1. Sequence Analysis, Purification, and Spectral Analysis of CYP153D17

**Figure 1:**
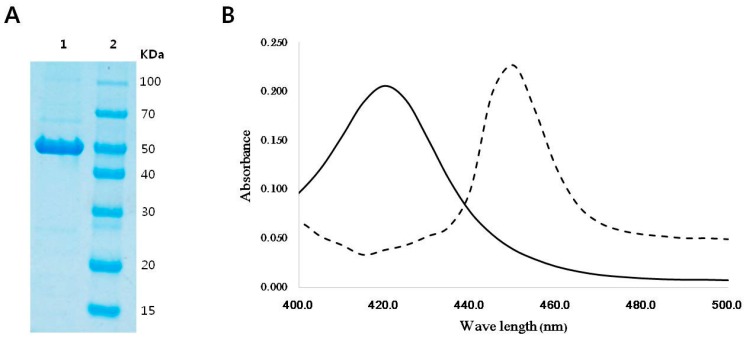
(**A**) Purification of CYP153D17: **Lane 1**, purified CYP153D17; **Lane 2**, molecular marker; (**B**) CO-reduced spectra of heterologously overexpressed CYP153D17. Oxidized form, solid line; CO-reduced form, dotted line.

CYP153D17 encodes a protein of 409 amino acids with an overall G + C content of 63.41%. Sequence analysis revealed that CYP153D17 has 48.27% identity with P450pyr hydroxylase from *Sphingopyxis macrogoltabida* and that it contains conserved motifs including an oxygen-binding domain and heme-binding domains found in typical CYPs [20]. When over-expressed heterologously in *Escherichia coli* C41 (DE3), as previously reported by our group [21], high levels of folded CYP153D17 were obtained. After purification by Ni^2+^-affinity chromatography, pure CYP153D17 was concentrated to 215.29 mg/mL. The molecular mass of CYP153D17 (48 kDa) was confirmed by sodium dodecyl sulfate polyacrylamide gel electrophoresis (SDS-PAGE) analysis and was consistent with the predicted molecular mass (Figure 1A). The ferric to ferrous reduction of CYP153D17 was achieved by the addition of sodium dithionite, and carbon monoxide was gassed through the sample tube. A shift of approximately 30 nm (from 420 to 450 nm) was observed in CYP153D17, indicating that the distribution of electron density on the heme was significantly perturbed (Figure 1B).

### 2.2. Substrate Binding Assay of CYP153D17

**Figure 2:**
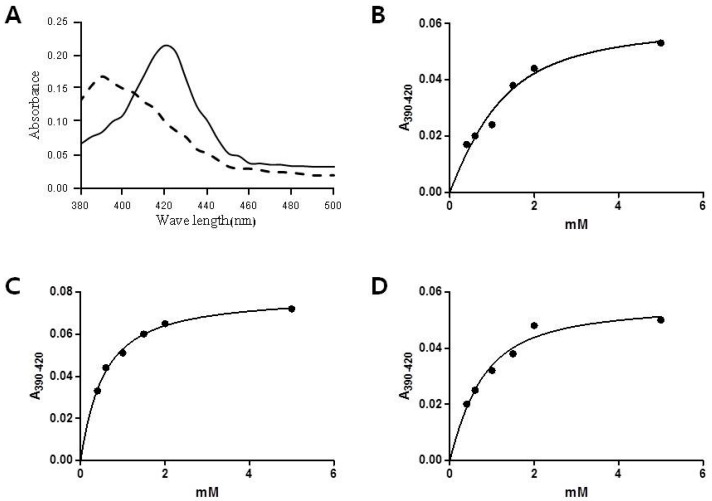

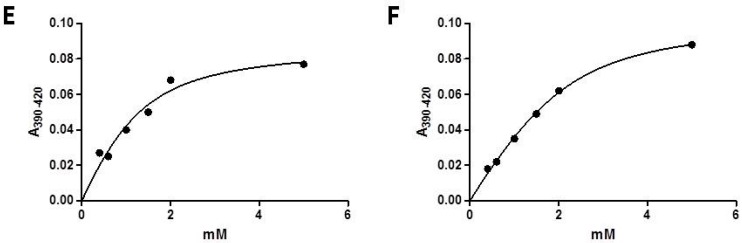
Spectral shift and titration curves of various alkanes and fatty acids. Differences in absorption were observed between A_390_ and A_420_ upon the addition of different concentrations (μM) of substrates and the fitted trace from which the *K_d_* value was determined. (**A**) Substrate-induced spin state shifts of a CYP153D17 substrate-free (solid line) and dodecane-bound (dotted line); (**B**) C10 alkane, decane; (**C**) C12 alkane, dodecane; (**D**) C10 fatty acid, capric acid; (**E**) C12 fatty acid, lauric acid; (**F**) C14 fatty acid, myristic acid.

To determine the binding affinity of CYP153D17, spectral binding titrations were performed with the alkanes (decane and dodecane) and fatty acids (capric acid, lauric acid, and myristic acid). All tested substrates changed the spectrum of CYP153D17, with a shift of the Soret band at 420 to 390 nm, which is typical of the high-spin form (Figure 2), after which the apparent dissociation constants from the titration curves (*K*_d_ values) were obtained (Table 1). Dodecane, capric acid, and lauric acid binding to CYP153D17 induced a ≥90% shift in the heme spin state from low-spin (Soret maximum at 420 nm) to high-spin (Soret maximum at 390 nm), whereas decane and myristic acid induced a ≥80% shift in the same heme spin state. In addition, the *K*_d_ values were 0.59 ± 0.69, 0.42 ± 0.14, 0.46 ± 0.41, 0.49 ± 0.70, and 0.54 ± 0.33 μM for decane, dodecane, capric acid, lauric acid, and myristic acid, respectively. Interestingly, although CYP153D17 showed different substrate affinities against C10–C12 alkanes and C10–C14 fatty acids, C12 alkane (dodecane) strongly bound to CYP153D17 with similar *K*_d_ values of C10–C12 fatty acids (capric acid and lauric acid).

### 2.3. Overall Structure of CYP153D17

**Figure 3:**
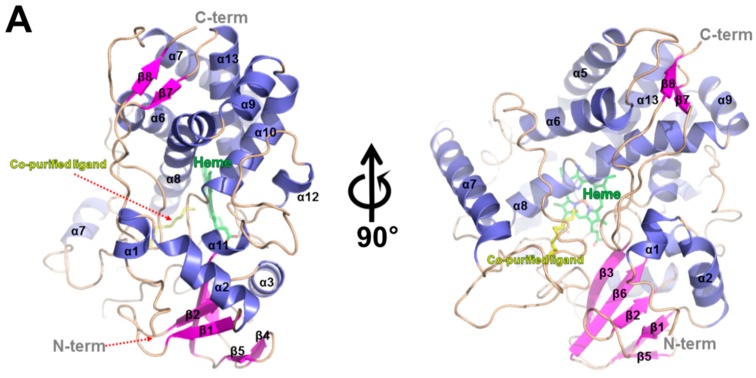

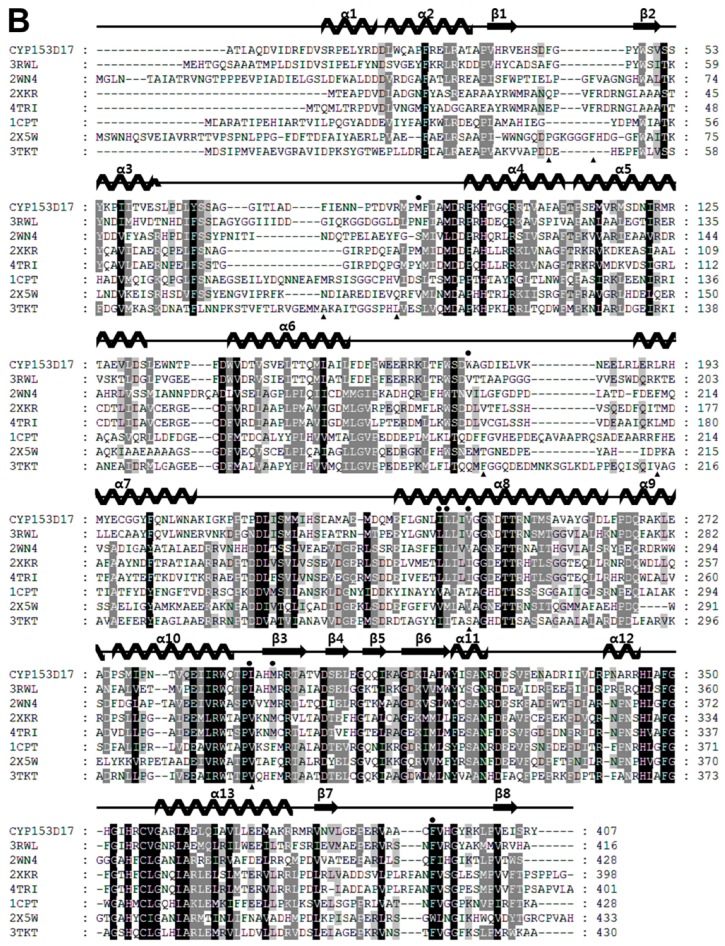
Crystal structure of CYP153D17. (**A**) The overall structure of CYP153D17 is shown as a ribbon diagram with α-helices colored slate blue and β-strands colored magenta. The bound heme molecule (green) and co-purified ligand (yellow) are shown in the stick model. N- and C-termini are labeled. The right-panel figure is rotated counterclockwise 90° from the left-panel figure; (**B**) Multiple sequence alignment of CYP153D17 (NCBI reference sequence, WP_010183267.1), P450pyr (PDB code, 3RWL; UniProtKB code, Q5F4D9), CYP124 (PDB code, 2WM4; UniProtKB code, P9WPP3), CYP142 (PDB code, 2XKR; UniProtKB code, P9WPL5), CYP142A2 (PDB code, 4TRI; UniProtKB code, A0R4Q6), P450terp (PDB code, 1CPT; UniProtKB code, P33006), CYP125 (PDB code, 2X5W; UniProtKB code, P9WPP1), and CYP108D1 (PDB code, 3TKT; UniProtKB code, Q2G3H6). Highly conserved residues are shaded in black and similar residues are in gray. The secondary structural elements in the crystal structure of CYP153D17 are shown above the multiple sequence alignment. The residues involved in binding of the co-purified ligand in CYP153D17 are indicated by a black circle above the sequence alignment, and those involved in phytanic acid binding in the CYP124 structure, by a black triangle below the sequence alignment. Multiple sequence alignment was performed using ClustalX (version 1.81) [22] and edited using GeneDoc (Ver 2.5.000) [23].

**Figure 4:**
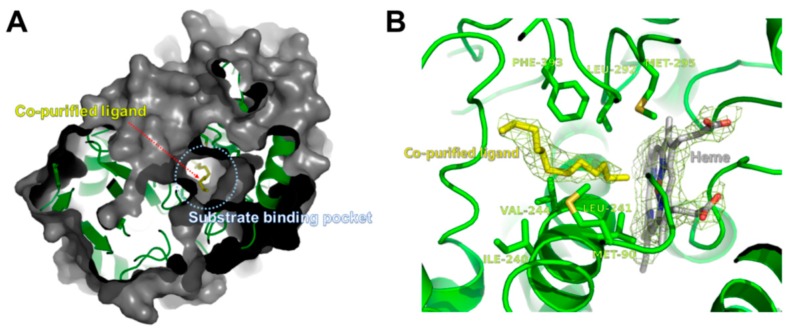
Co-purified ligand in the substrate-binding pocket of CYP153D17. (**A**) The co-purified ligand molecule is represented as a yellow stick, along with the surface representation of CYP153D17; (**B**) The CYP153D17 residues involved in interactions with the co-purified ligand are represented by green sticks. 2Fo-Fc electron density map at 1.0 σ was shown around co-purified dodecane-like molecule and heme group.

The crystal structure of CYP153D17 from *Sphingomonas* sp. strain PAMC 26605 was determined by molecular replacement (MR) performed using the P450pyr hydroxylase structure from *S. macrogoltabida* (Protein Data Bank code, 3RWL) as a search model. The structure was refined to a resolution of 3.1 Å (Table 2) and had a typical CYP fold, comprising a central heme molecule surrounded by 13 α-helices and 7 β-strands (Figure 3). The putative substrate-binding pocket of CYP153D17 was found to be shaped similar to a cone funnel with a length and outer diameter of approximately 17 and 10 Å, respectively (Figure 4A). Notably, an additional electron density map appeared within the substrate-binding pocket during refinement. Because exogenous ligands were not added to the crystallization solutions, the bound molecule may have co-purified with the enzyme during the production of recombinant protein. The resulting electron density map revealed the presence of a long fatty acid or alkane-like compound. Substrate binding assay data suggested that the ligand was an alkane-like compound and the electron density was modeled as dodecane. Although dodecane was modeled as a putative ligand bound to CYP153D17, the actual substrate was not determined. The co-purified ligand was bound to the hydrophobic substrate-binding pocket created by the Met90, Ala175, Ile240, Leu241, Val244, Leu292, Met295, and Phe393 residues (Figure 4B).

Next, a structural homology search was performed using the DALI server [24]. The top six unique hits were identified and are shown in Table 3. The results showed that CYP153D17 had the highest degree of structural similarity with P450pyr (PDB code, 3RWL) [13] and that CYP153D17 shared structural similarity with CYP124 (PDB code, 2WM4) [25], CYP142 (PDB code, 2XKR) [26], P450terp (PDB code, 1CPT) [27], CYP125 (PDB code, 2X5W) [28], and CYP108D1 (PDB code, 3TKT) [29]. Sequence alignments of these proteins revealed that several residues constituting the hydrophobic substrate-binding pocket of CYP153D17 were highly conserved. Specifically, Ile240, Leu292, and Phe393 were found to be highly conserved, while other residues (Met90, Ala175, Leu241, Val244, and Met295) were less conserved (Figure 3B).

### 2.4. Structural Comparisons of CYP153D17 with Other CYPs

**Figure 5:**
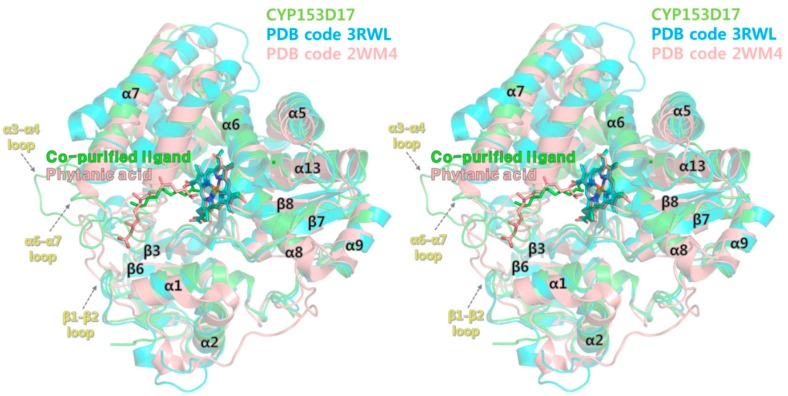
Stereo view of the crystal structures of P450pyr (PDB code, 3RWL; blue) and phytanic acid–bound CYP124 (PDB code, 2WM4; salmon) superimposed on the CYP153D17 structure (green). Secondary structure elements were named based on the CYP153D17 structure.

The overall protein structure of P450pyr (best hit in the DALI search) was very similar to that of CYP153D17 (r.m.s. deviation, 0.96 Å for 307 Cα atoms), but P450pyr contained no ligand and the loop region (residues 88–96 in P450pyr) between the α3 and α4-helix was missing in the P450pyr structure (Figure 5). This loop may be more flexible in the absence of substrate in P450pyr. Structural comparison of CYP124 (second hit in the DALI search) with CYP153D17 showed overall structural similarity in the core region structure (r.m.s. deviation, 1.24 Å for 327 Cα atoms), whereas considerable local conformational differences were observed in the β1–β2, α3–α4, and α6–α7 connecting loop regions (in secondary structure annotation of CYP153D17). The CYP124 structure was complexed with phytanic acid, with hydrophobic interactions involving Leu60, Phe63, Asn97, Phe107, Leu198, Phe212, Val266, and Val315 within 4 Å distance. The mapping of interaction residues onto the sequence alignment showed that the substrate-binding residues differed considerably between CYP153D17 and CYP124 (Figure 3B). Structural superposition revealed that the binding mode of phytanic acid was similar to that of the co-purified ligand of CYP153D17 as an extended conformation. This finding suggests mechanical-reaction-related similarities between CYP153D17 and CYP124. CYP124 catalyzes ω-oxidation of phytanic acid; that is, the terminal methyl group of phytanic acid is specifically hydroxylated. Therefore, it is expected that for CYP153D17, hydroxylation may also be performed at the end of alkane molecules, such as in CYP124-mediated ω-oxidation.

## 3. Materials and Methods

### 3.1. Cloning, Over-Expression, and Purification

A set of primers, CYP153D17F (5′-GGA TCC ATT ATG GCG ACT CTG GCG-3′) (*Bam*HI, forward primer) and CYP153D17R (5′-AAG CTT AAC TGA GCG TGC TCA ATA GCG-3′) (*Hin*dIII, reverse primer), was used to amplify CYP153D17. Polymerase chain reaction (PCR) was performed in a thermocycler (Takara, Shiga, Japan). The amplification conditions were 94 °C for 5 min, followed by 35 cycles of each of the following conditions: 94 °C for 1 min, 60 °C for 1 min, 72 °C for 1.5 min, and 72 °C for 7 min. The 20-μL PCR mixture contained 10 μL PCR Mix (Noble Bio, Hwaseoug-Si, Korea), 1 μL forward primer, 1 μL reverse primer, 1 μL template DNA, and 7 μL distilled water. The obtained PCR product (1236 bp) was cloned into the same restriction enzyme sites as in pET-28a(+) to construct pN153D17, and recombinant pN153D17 was introduced into *Escherichia coli* C41 (DE3) by heat-pulse transformation.

For overexpression, *E. coli* transformants harboring pN153D17 were cultured in LB (Luria-Bertani) medium with an appropriate amount of kanamycin, and the culture was grown at 37 °C until cell density reached 0.6–0.8 at OD_600_. Next, 1 mM 5-α-aminolevulinic acid and 0.5 mM FeCl_3_ were added to the culture. After 30-min incubation at 20 °C, protein overexpression was induced by adding 0.5 mM IPTG (isopropyl-β-d-1-thiogalactopyranoside) followed by incubation for 70 h at 20°C. *Escherichia coli* cultures were harvested and washed twice with 50 mM Tris-HCl buffer with 10% glycerol (pH 7.4), and finally lysed by an ultrasonicator. After 12,000-rpm centrifugation for 25 min at 4 °C, the obtained soluble proteins were purified by metal affinity with Ni^2+^–NTA resin (Qiagen, Hilden, Germany). The eluted CYP153D17 protein was concentrated using Amicon Ultra centrifugal filters (Ultracel-3K; Millipore, Billerica, MA, USA) and confirmed by 15% SDS-PAGE analysis. After the poly-His tag was cleaved by thrombin by overnight incubation at 4 °C, CYP153D17 was loaded onto a Superdex-200 column (GE Healthcare, Little Chalfont, UK) that had been equilibrated with 50 mM potassium phosphate and 100 mM sodium chloride, pH 7.4. The fractions containing red-colored CYP153D17 were collected and concentrated to 215.29 mg/mL by centrifugal filtration.

### 3.2. Substrate Binding Assay

To determine whether CYP153D17 can bind alkanes and fatty acids, a UV-Vis (Ultraviolet-Visible) spectral binding titration assay was performed. Binding of the substrate to CYP153D17 induced a shift of the Soret band of the CYP absorption spectrum from A_420_ to A_390_ [30]. Alkanes (decane and dodecane) and fatty acids (capric acid, lauric acid, and myristic acid) were dissolved in ethanol to prepare stock solutions of different concentrations (0.4–5 mM) for each substrate. Binding of substrate to CYP153D17 was carried out by the consecutive addition of 1-μL aliquots from the stock solutions to 1 mL of 1 μM CYP153D17 solution, and the UV–Vis spectra of the mixture were recorded. To calculate *K*_d_, the difference between adsorption at A_390_ and A_420_ was calculated, corrected with the A_390_-to-A_420_ value at 0 M substrate, and plotted against the substrate concentrations. The data were fitted to equations as described by Funhoff et al. [31].

### 3.3. Crystallization and Data Collection

Initial crystallization screening was performed using the sitting-drop vapor-diffusion method with a Mosquito crystallization robot (TTP Labtech, Melbourn, UK) and several commercially available screening kits such as MCSG I-IV (Microlytic, Burlington, MA, USA), SaltRx, Index (Hampton Research, Aliso Viejo, CA, USA), and SG1 Screen (Molecular Dimensions, Suffolk, UK). Each crystallization drop consisted of 200 nL protein solution and 200 nL reservoir solution against 80 µL reservoir solution in 96-well sitting-drop plates (Emerald Bio, Bainbridge Island, WA, USA) at 293 K. CYP153D17 was crystallized under several different conditions within two days. To obtain optimal crystals, the crystallization condition of 25% (*w*/*v*) PEG 3350 (SG-1 #E2) was varied according to the precipitant concentration by using the hanging-drop vapor-diffusion method in 24-well crystallization plates (Molecular Dimensions) at 293 K. The drop volume was increased to 1 µL protein and 1 µL reservoir solution against 500 µL reservoir solution. An optimal single crystal was obtained using 22% (*w*/*v*) PEG 3350 and had a rectangular stick shape. The single crystal was picked using a loop and mounted without cryoprotectant. Diffraction data were collected using a synchrotron-radiation source at the BL-7A beam line, Pohang Accelerator Laboratory, Pohang, Korea.

### 3.4. Structure Determination and Refinement

The 3.1 Å resolution data set contained 100 images with a detector-to-crystal distance of 300 mm, exposure time of 1 s, and oscillation range of 1° per image. The data set was indexed, integrated, and scaled using HKL-2000 software [32]. The data collection statistics are summarized in Table 2. For structure determination of CYP153D17, MR was performed using the program MOLREP in the CCP4 suite [33,34]. The coordinate of P450pyr hydroxylase from *S. macrogoltabida* (PDB code, 3RWL) was used for MR searching. The structure model of CYP153D17 was iteratively rebuilt and refined by manually fitting the residues and backbone into the electron density map by using the WinCoot and REFMACK programs from the CCP4 suite [35,36]. The quality of the refined structure was validated using the MOLPROBITY server and SFCHECK with an *R*_work_ of 26.25% and *R*_free_ of 34.07% [37,38]. The statistical data for structure refinement are provided in Table 2.

## 4. Conclusions

As major components of natural gas and petroleum, alkanes play important roles as energy and sole carbon sources in modern human life. However, because of their solid viscosity and inertness in the soil and water bodies resulting from accidental oil spills and leakage, many scientists have recently considered alkane-degrading microorganisms with various uptake mechanisms and enzyme systems to be a solution to these issues [4,5]. One of these enzyme systems, alkane hydroxylase, is quite efficient in oxidizing various substrates in industrial applications. In this context, the chemistry of bacterial alkane hydroxylase is interesting and rare. Among the structurally available bacterial alkane hydroxylases, P450pyr is the only member of the CYP153A family with alkane function [13], and this is the first study to determine structural information for the CYP153D family of alkane hydroxylases. In this study, we identified CYP153D17 from the endosymbiotic bacterium *Sphingomonas* sp. PAMC 26605 as a putative alkane-hydroxylating cytochrome P450 enzyme based on our substrate binding assay and structural data. However, we cannot exclude the possibility that CYP153D17 may have also fatty-acid hydroxylation activity considering its broad substrate specificity. Thus, further investigations are needed to identify true intrinsic ligands of CYP153D17. Nevertheless, our substrate binding assay and combined structural studies provide several insights into the molecular basis of substrate binding by CYP153D17 and its regio- and stereo-selectivity towards various substrates containing alkanes and fatty acids. In addition, our results will facilitate future studies identifying active-site residues and investigating their roles in different substrate (alkane and fatty acid) specificities. Our results may also provide a structural basis for protein engineering.

## Figures and Tables

**Table 1:** Binding Constants of Purified CYP153D17 with Various Alkanes and Fatty Acids.

Substrate	Spin Shift (%)	Apparent *K*_d_ Value (μM)
Decane	≥80	0.59 ± 0.69
Dodecane	≥90	0.42 ± 0.14
Capric acid	≥90	0.46 ± 0.41
Lauric acid	≥90	0.49 ± 0.70
Myristic acid	≥80	0.54 ± 0.33

**Table 2:** X-ray data collection and refinement statistics.

Data Set	CYP153D17 Complexed with Dodecane
Data collection	
X-ray source	PLS-7A
Space group	P3121
Unit-cell parameters (Å)	a = 110.837, b = 110.837, c = 113.18
Wavelength (Å)	0.97933
Resolution Range (Å)	50.00–3.10 (3.15–3.10)
No. of observed reflections	62,438
No. of unique reflections	14,358
Completeness (%)	94.3 (97.2)
Redundancy	4.4 (4.5)
*R*_merge_^a^	0.093 (0.501)
*I/σ*	26.8 (4.9)
Refinement	
Resolution range	50.00–3.10 (3.18–3.10)
Reflections: working/free	13,128/689 (981/43)
*R*_cryst_^b^	0.261 (0.430)
*R*_free_^c^	0.340 (0.463)
Ramachandran plot: favored/allowed/disallowed (%) ^d^	75.1/98.0/22.9
R.m.s.d. bonds (Å)	0.0126
R.m.s.d. angles (°)	1.7974
PDB accession code	5H1Z

^a^ *R*_merge_ = ∑|<I> − I|/∑<I>; ^b^ *R*_cryst_ = ∑||Fo| − |Fc||/∑|Fo|; ^c^ *R*_free_ calculated with 5% of all reflections excluded from refinement stages using high-resolution data; ^d^ The Ramachandran plot was calculated by MolProbity (http://molprobity.biochem.duke.edu/); Values in parentheses indicate the statistics of the highest resolution shells. R.m.s.d. is the abbreviation for the root mean square deviation.

**Table 3:** Selected structural homologues of CYP153D17 from a DALI search (DALI-Lite server ^a^).

Protein	PDB Code	DALI Score	Ligand	Sequence % ID with CYP153D17 (Aligned Residue no./Total Residue no.)	Reference
P450pyr	3RWL	47.6	NL ^b^	47% (361/404)	[13]
CYP124 (Methyl-branched lipid ω-hydroxylase)	2WM4	44.7	Phytanic acid	30% (365/426)	[25]
CYP142 (Cholesterol oxidase)	2XKR	44.6	Tetraethylene glycol	28% (359/395)	[26]
P450terp	1CPT	44.5	NL	27% (366/412)	[27]
CYP125	2X5W	44.0	Cholest-4-en-3-one	27% (366/409)	[28]
CYP108D1	3TKT	43.6	NL ^b^	29% (367/411)	[29]

^a^ DaliLite version 3 server, http://ekhidna.biocenter.helsinki.fi/dali_server/; ^b^ NL, No ligand molecule is bound.
